# Enhanced cognitive behaviour therapy for adolescents with anorexia nervosa: An alternative to family therapy?

**DOI:** 10.1016/j.brat.2012.09.008

**Published:** 2013-01

**Authors:** Riccardo Dalle Grave, Simona Calugi, Helen A. Doll, Christopher G. Fairburn

**Affiliations:** a Department of Eating and Weight Disorders, Villa Garda Hospital, Via Montebaldo, 89, I-37016 Garda (VR), Italy; b Norwich Medical School, University of East Anglia, Norwich NR4 7TJ, UK; c Oxford University, Department of Psychiatry, Warneford Hospital, Oxford OX3 7JX, UK

**Keywords:** Anorexia nervosa, Treatment, Cognitive behaviour therapy, Eating disorder, Family therapy

## Abstract

A specific form of family therapy (family-based treatment) is the leading treatment for adolescents with anorexia nervosa. As this treatment has certain limitations, alternative approaches are needed. “Enhanced” cognitive behaviour therapy (CBT-E) is a potential candidate given its utility as a treatment for adults with eating disorder psychopathology. The aim of the present study was to establish, in a representative cohort of patients with marked anorexia nervosa, the immediate and longer term outcome following CBT-E. Forty-nine adolescent patients were recruited from consecutive referrals to a community-based eating disorder clinic. Each was offered 40 sessions of CBT-E over 40 weeks from a single therapist. Two-thirds completed the full treatment with no additional input. In these patients there was a substantial increase in weight together with a marked decrease in eating disorder psychopathology. Over the 60-week post-treatment follow-up period there was little change despite minimal subsequent treatment. These findings suggest that CBT-E may prove to be a cost-effective alternative to family-based treatment.

## Highlights

► CBT-E is designed to address eating disorder psychopathology in adults. ► The present findings show that CBT-E can also be used with adolescents. ► Almost two-thirds of the adolescent patients completed CBT-E. ► Among the completers there was a substantial, and well maintained, response. ► CBT-E may be a cost-effective alternative to family-based treatment.

## Introduction

Anorexia nervosa has a profound impact on physical health and psychosocial functioning. It is important to treat it early and effectively as otherwise it can have long-lasting effects. A particular form of family therapy, termed family-based treatment (FBT, Lock, Le Grange, Agras, & Dare, 2001), is the leading empirically-supported intervention for adolescents with the disorder (NICE, 2004).

FBT is not without limitations. It is not acceptable to some families and patients; it is labour intensive and therefore costly; and fewer than half the patients make a full treatment response (Lock, 2011; Lock et al., 2010). FBT therefore needs to be modified to make it more acceptable and effective, or alternative treatment approaches need to be found (Lock, 2011).

Cognitive behaviour therapy is a potential candidate as an alternative to FBT (Lock et al., 2010). It is the leading empirically supported treatment for bulimia nervosa (NICE, 2004; Shapiro et al., 2007), a disorder with psychopathology that overlaps with that of anorexia nervosa. Furthermore, the treatment has been adapted to make it suitable for any form of eating disorder, including anorexia nervosa (Fairburn, 2008b; Fairburn, Cooper, & Shafran, 2003). The new “enhanced” form of the treatment (CBT-E) has been found in two independent studies (combined *N* = 245) to produce sustained change in those eating disorder patients who are not significantly underweight (i.e., those with bulimia nervosa or eating disorder not otherwise specified (Byrne, Fursland, Allen, & Watson, 2011; Fairburn et al., 2009). It has also been shown to be associated with a good outcome in two cohorts of adults with anorexia nervosa (total *N* = 99) (Fairburn, Cooper, Doll, Palmer, & Dalle Grave, (2013)) and in a cohort of severely affected inpatients (*N* = 80) (Dalle Grave, Calugi, Conti, Doll, & Fairburn, submitted for publication). However, its utility with adolescents has yet to be established.

The overall aim of the present study was to provide benchmark data on the effects of CBT-E on adolescents with anorexia nervosa and to determine whether it might be a viable alternative to FBT. The specific aim was to address three key clinical questions. First, among adolescents with anorexia nervosa, what proportion is able to complete CBT-E without the need for additional treatment? Second, among the patients who do complete CBT-E, what is their outcome? Third, how well are the changes maintained following CBT-E?

## Method

### Design

A cohort of adolescent patients with anorexia nervosa was recruited from consecutive referrals to an eating disorder clinic. Eligible patients were offered 40 sessions of CBT-E over 40 weeks. This was to be their sole psychological intervention. Those patients who agreed received this treatment and were then followed-up 60 weeks later. The study was approved by the local human subjects committee.

### Setting and participants

The sample was recruited from consecutive referrals by family doctors and other clinicians to a well-established eating disorder clinic serving the Verona area of Italy. The patients had to be aged between 13 and 17 years and to fulfil the DSM-IV diagnostic criteria for anorexia nervosa (Association, 1994) bar the amenorrhoea criterion. In addition, the patient's parents or legal guardians had to provide written informed consent to their participation after having received a full description of the study. The exclusion criteria were as follows: i) being unsafe to manage on an outpatient basis (*N* = 2); ii) having received in the previous year a specialist treatment for anorexia nervosa (*N* = 1); iii) having a co-existing Axis 1 psychiatric disorder that precluded immediate eating disorder-focused treatment (e.g., psychosis or drug dependence, *N* = 2); and iv) not being available for the 40 week period of treatment (*N* = 2). The aim was to recruit a sample of 50 patients so that the study had 80% power to detect a moderate change from baseline equivalent to an effect size of around 0.4.

### The treatment

CBT-E is a treatment for people with eating disorder psychopathology, irrespective of their eating disorder diagnosis. It is primarily an outpatient-based treatment although a version for inpatients has been devised and evaluated (Dalle Grave, 2011; Dalle Grave et al., submitted for publication.). A detailed guide to the implementation of CBT-E has been published (Fairburn, 2008a) that specifies the adaptations for adolescents (Cooper & Stewart, 2008).

With adults who are underweight, CBT-E has three phases. In the first, the emphasis is on helping patients think afresh about their current state and the processes maintaining it. This is followed by a detailed analysis of the pros and cons of tackling their eating disorder. Then, if willing, patients are helped to regain weight while at the same time they address their eating disorder psychopathology and the processes maintaining it. Particular emphasis is placed upon the modification of the concerns about shape and weight. In the final phase the focus is on helping patients maintain the changes that they have made. This includes developing personalised strategies for the rapid correction of setbacks.

The same treatment approach is used with adolescents. The only significant difference is the routine involvement of the patient's parents. With adult patients significant others are involved if this will facilitate the one-to-one treatment. The same applies to the treatment of adolescent patients except that the parents are invariably required to facilitate treatment.

In the present study CBT-E comprised 40, 45-min, one-to-one sessions over 40 weeks, preceded by two 60-min preparatory sessions and followed by one review session 20 weeks after the end of treatment. The majority of the sessions were attended by the adolescent patient alone. Parental involvement consisted of a single 1-h assessment session during the first two weeks of treatment and eight 15-min sessions with the patient and parents together. These took place immediately after an individual session with the patient, and occurred at weeks 1–4 and at weeks 8, 12, 20, and 40. The aim of the initial session with parents was to identify family factors liable to hinder the patient's attempts to change while the subsequent sessions were devoted to meal planning, the conduct of mealtimes and to the generation of solutions to problems that had emerged or were foreseeable. Additional sessions with the parents only took place if there were family crises, extreme difficulties at mealtimes or parental hostility towards the adolescent. Few such sessions were needed.

A single therapist treated each patient with a substitute stepping in if the primary therapist had to be absent. The patients had no additional therapeutic input, either from physicians, dieticians or other health professionals unless there was a specific indication (e.g., the management of medical complications or comorbid conditions).

### Assessment

The main assessment points were before treatment, at the end of treatment and 60 weeks later.

#### Body weight and body mass index

Weight was measured using a beam balance scale and height was measured using a wall-mounted stadiometer. Body mass index (BMI) centiles were calculated using the Center for Disease Control and Prevention growth charts (www.cdc.gov/growthcharts).

#### Eating disorder features

The Italian version of the self-report Eating Disorder Examination Questionnaire (EDE-Q6.0) was used (Fairburn & Beglin, 2008).

#### General psychiatric features

The full version of the Symptom Checklist-90 was used from which a Global Severity Index (GSI) was calculated (Derogatis & Spencer, 1982).

### Statistical analysis

The statistical analysis was undertaken by HAD using standard treatment research data analytic procedures. Data are presented as *N* (%) for categorical data and as means (with standard deviation, SD) or medians (with range) for continuous data.

## Results

### The sample

Forty-nine eligible patients were offered CBT-E and 46 accepted. Their mean age was 15.5 years (SD 1.3, range 13–17 years) and all were female, white and single. The mean duration of the eating disorder was 0.86 years (range 0–5, median 0.5 years). The patients were substantially underweight, with 23 (50%) having a BMI centile of <1 and the mean (SD) BMI centile (taking BMI centile as 0.5 for those with a value < 1) being 2.86 (SD 3.35, range 0.5–13.0, median 0.75).

### Intent-to-treat findings at end of treatment and 60-week follow-up

Although the primary goal of this study was to determine the proportion of patients that can complete this outpatient treatment, and their treatment response, intent-to-treat data are reported in Table 1. Given the poor treatment response in anorexia nervosa, we chose to use a highly conservative method of data imputation, namely moving the initial data point forward. Even using this method, there was a marked increase in weight. By the end of treatment the mean BMI centile had increased from 2.86 (SD 3.35) to 19.8 (SD 19.2) and over the 60-week period of follow-up it had increased still further (mean BMI centile 23.6, SD 25.7). At this point 28.3% (13/46) of the patients had gained sufficient weight from baseline to reach 95% of their expected weight. The increase in weight was accompanied by a decrease in eating disorder psychopathology and general psychiatric features.

### Question 1 – What proportion of patients complete CBT-E?

Almost two-thirds of the patients completed the full 40 sessions of CBT-E without any additional treatment (*n* = 29, 63.0%). Patients who required additional input, either because of concerns about their physical or psychological state or because of sustained lack of progress, were classed as non-responders (*n* = 9, 19.6%) as were those who ceased to attend (*n* = 8, 17.4%).

### Question 2 – What is the outcome among those who complete CBT-E?

There was a substantial increase in weight among the 29 treatment completers (see Table 2). The mean weight gain was 8.60 kg (SD 4.14; 95% CI 7.03 to 10.18; *p* < 0.001), equivalent to a BMI centile increase of 27.0 (SD 15.5; 95% CI 21.1–32.9). One third (32.1%, 9/28) gained sufficient weight to reach 95% of the expected weight for their age and sex. Eating disorder psychopathology and general psychiatric features improved substantially with the mean global EDE-Q score decreasing by 2.03 (SD 1.25; 95% CI 1.55 to 2.50; *p* < 0.001) and the mean GSI decreasing by 0.67 (SD = 0.63; 95% CI 0.43 to 0.91; *p* < 0.001). Almost all the patients (96.6%, *n* = 28/29) had minimal residual eating disorder psychopathology, defined as having a global EDE-Q score below 1SD above the community mean (i.e., <2.77).

### Question 3 – Are the changes sustained following CBT-E?

There was high compliance with follow-up with 100% (29/29) of the treatment completers being reassessed at 60-week follow-up. Few had required additional treatment in the interim: three had received further treatment, and four were given one to five brief CBT-E “booster” sessions.

Overall the changes made during treatment were well maintained (see Table 2). Weight gain continued with the mean weight increasing by 2.22 kg (SD 7.4) from the end of treatment to follow-up and the BMI centile rising from a mean (SD) of 29.4 (16.2) at the end of treatment to 33.7 (25.4) at follow-up. A further four patients reached 95% of the expected weight for their age and sex resulting in 44.8% (*n* = 13/29) of the treatment completers meeting this criterion. Almost ninety percent (26/29, 89.7%) had minimal residual eating disorder psychopathology as defined above.

## Discussion

The aim of the present study was to provide benchmark data on CBT-E in order to determine whether it might be a potential alternative to FBT for adolescents with marked anorexia nervosa. To achieve this aim, consecutive referrals to an eating disorder clinic were treated with CBT-E and then followed-up 60 weeks later.

There were three main findings. The first was that two-thirds of the patients were able to complete this lengthy outpatient treatment without the need for additional input. The remaining third were classed as non-responders, either because they had to have additional treatment due to concerns about their physical health or lack of progress, or because they ceased to attend.

The second finding was that among those who completed CBT-E there were substantial improvements in weight and eating disorder psychopathology. The mean BMI centile increase was 26.9 with a third of the patients gaining sufficient weight reach 95% of their expected weight. In addition, there were substantial reductions in eating disorder and general psychopathology.

The third finding concerns the stability of the changes obtained. These were well maintained over 60 weeks despite minimal exposure to additional treatment. This is in contrast to FBT where additional treatment is common during follow up and is likely to contribute to the reported outcome.

The present findings are promising. Given the size and severity of the sample, the magnitude of the response and the fact that it was well maintained, there is now a compelling case for comparing CBT-E and FBT in randomised controlled trials. Key variables of interest would include the relative acceptability of the two approaches, their effectiveness and their ability to produce enduring change. Their relative cost and cost-effectiveness would also be important to determine. Both treatments are readily disseminated. The fact that CBT-E can also be used with adults and with all forms of eating disorder is a strength of the approach.

In addition to determining the relative effects of the two treatments, it would also be of theoretical interest and practical importance to determine whether there are moderators of treatment response that might allow the matching of patients to CBT-E or FBT. This is not implausible as they differ markedly in their strategies, procedures and postulated modes of action. While FBT has an “atheoretical” stance (Lock, 2011; Loeb, Lock, le Grange, & Greif, 2012), the nature of the treatment suggests that it is best suited to the needs of the younger adolescent patient. In contrast, CBT-E is designed to modify the mechanisms that maintain eating disorder psychopathology (Fairburn, 2008b; Fairburn et al., 2003) and it might therefore be expected to show its greatest effects in those adolescent patients in whom these processes are operating. Such patients will have a more enduring eating disorder and therefore be older than the average adolescent patient. The older patients may also find an “adult” form of treatment more acceptable than a family style one. These predictions need testing.

## Funding

CGF is supported by a Principal Research Fellowship from the Wellcome Trust (046386). HAD is supported by a programme grant from the Wellcome Trust (046386). The Wellcome Trust had no involvement in the design or execution of this study.

## Figures and Tables

**Table 1:** Clinical characteristics before treatment, after treatment and at 60-week follow-up among all patients (intent to treat data set, *n* = 46). Data are shown as mean (SD) unless otherwise stated.

	Before treatment	After treatment	At 60-week follow-up
Weight			
Body weight (kg)	40.0 (5.7)	45.5 (7.4)***	48.4 (9.4)***
Body mass index centile	2.86 (3.35)	19.8 (19.2)***	23.6 (25.7)***
Weight 95% of that expected, *n* (%)	1 (2.2)	10 (21.7)**	13 (28.3%)***
Eating disorder psychopathology			
Overall severity (global EDE-Q)	2.79 (1.5)	1.50 (1.5)***	1.51 (1.6)***
Global EDE-Q < 1SD above the community mean,^a^*n* (%)	18 (41.9%)	34 (79.1%)***	33 (76.7%)***
Dietary restraint (EDE-Q subscale)	2.69 (1.8)	1.37 (1.7)***	1.47 (1.9)***
Eating concern (EDE-Q subscale)	2.58 (1.5)	1.34 (1.5)***	1.28 (1.6)***
Shape concern (EDE-Q subscale)	3.17 (1.7)	1.92 (1.7)***	1.86 (1.9)***
Weight concern (EDE-Q subscale)	2.75 (1.6)	1.49 (1.7)**	1.45 (1.5)***
Eating disorder behaviour (EDE-Q)			
Binge eating, *n* (%) present	2 (4.3%)	1 (2.2%)	9 (19.6%)
if present, episodes/28 days, median (range)	38.5 (7–70)	70	6 (1–70)
Self-induced vomiting, *n* (%) present	1 (2.2%)	1 (2.2%)	2 (4.3%)
if present, episodes/28 days, median(range)	120	120	65 (10–120)
Laxative misuse, *n* (%) present	1 (2.2%)	1 (2.2%)	1 (2.2%)
If present, episodes/28 days, median (range)	12	12	8
General psychiatric features, GSI	1.18 (0.6)	0.74 (0.6)***	0.68 (0.6)***

EDE-Q – Eating Disorder Examination Questionnaire (version 6.0) (Fairburn & Beglin, 2008). GSI – Global Severity Index (Derogatis, 1977; Derogatis & Spencer, 1982). *p < 0.05; **p < 0.01; ***p < 0.001 vs baseline.

a Global EDE-Q less than 1SD above community EDE-Q mean for young adult women (Mond, Hay, Rodgers, & Owen, 2006) (i.e., <2.77).

**Table 2:** Characteristics before treatment, after treatment and at 60-week follow-up among those (*n* = 29) who completed CBT-E. Data are shown as mean (SD) unless otherwise stated.

	Before treatment	After treatment	At 60-week follow-up
Weight			
Body weight (kg)	41.0 (5.3)	49.6 (4.3)***	50.9 (5.5)***
Body mass index centile	3.36 (3.73)	30.3 (16.7)***	35.1 (26.0)***
Weight 95% of that expected, *n* (%)	1 (3.4)	10 (34.5%)***	13 (44.8%)***
Eating disorder psychopathology			
Overall severity (global EDE-Q)	2.87 (1.4)	0.84 (1.0)***	1.01 (1.3)***
Global EDE-Q < 1SD above the community mean,^a^*n* (%)	11 (37.9%)	28 (96.6%)***	26 (89.7%)***
Dietary restraint (EDE-Q subscale)	2.71 (1.6)	0.71 (0.9)***	0.83 (1.2)***
Eating concern (EDE-Q subscale)	2.67 (1.5)	0.66 (0.9)***	1.32 (1.6)***
Shape concern (EDE-Q subscale)	3.22 (1.6)	1.27 (1.3)***	0.83 (1.3)***
Weight concern (EDE-Q subscale)	2.91 (1.6)	0.72 (1.1)***	1.05 (1.4)***
Eating disorder behaviour (EDE-Q)			
Binge eating, *n* (%) present	1 (3.4%)	0	5 (17.2%)
If present, episodes/28 days, median (range)	7	–	6 (1–15)
Self-induced vomiting, *n* (%) present	0	0	0
If present, episodes/28 days, median(range)	–	–	–
Laxative misuse, *n* (%) present	0	0	0
If present, episodes/28 days, median(range)	–	–	–
General psychiatric features, GSI	1.18 (0.6)	0.51 (0.4)***	0.48 (0.4)***

EDE-Q – Eating Disorder Examination Questionnaire (version 6.0) (Fairburn & Beglin, 2008). GSI – Global Severity Index (Derogatis, 1977; Derogatis & Spencer, 1982). *p < 0.05; **p < 0.01; ***p < 0.001 vs baseline.

a Global EDE-Q less than 1SD above community EDE-Q mean for young adult women (Mond et al., 2006) (i.e., <2.77).
